# High Education and University Teaching and Learning Processes: Soft Skills

**DOI:** 10.3390/ijerph191710699

**Published:** 2022-08-27

**Authors:** Antonio Ragusa, Valeria Caggiano, Rubén Trigueros Ramos, Jerónimo J. González-Bernal, Ana Gentil-Gutiérrez, Susana Adelina Moreira Carvalho Bastos, Josefa González-Santos, Mirian Santamaría-Peláez

**Affiliations:** 1 Rome Business School, Department of Education, 00196 Rome, Italy; 2 Department of Education, University Roma TRE, 00154 Rome, Italy; 3 Hum-878 Research Team, Health Research Centre, Department of Psychology, University of Almería, 04120 Almería, Spain; 4 Department of Health Sciences, University of Burgos, 09001 Burgos, Spain; 5 ISCAP–CEOS.PP, Porto Polytechnic School, Accounting Department, 4200-465 Porto, Portugal

**Keywords:** teaching competences, soft skills, cultural values, European education system

## Abstract

In the era of globalization and technology, society demands young generations of citizens able to work in a kind of environment characterized by complexity and diversity. Therefore, the education system faces a new kind of challenge, as graduates are reported to need transversal skills, which are unlikely to be learned through traditional classroom teaching. The overall aim of this article is to examine the needs for these skills and their acquisition by higher education teachers to cope with the evolving European labour market of the 2020s. The article envisions the importance of soft skills in the teaching profession. The empirical part consists of a comparative study with Italian and Portuguese teachers, highlighting the status of a set of crucial soft skills (assertiveness, networking skills, teamwork, sensitivity, socialization, action-orientation, ability to work under pressure and social desirability).

## 1. Introduction

Particular importance has been attached to the quality of teaching in higher education in recent years, both nationally and internationally. In the European Union, the urgent need for improvement of the quality and status of teaching and the modernization of higher education has been highlighted [1,2]. In this process, teachers’ competences play an essential role and it is important that future teachers are prepared to work with complex content and an increasing diversity of learners [3,4]. It should therefore be a priority for teachers to start with high-quality initial professional preparation [5]. It is also conceivable that teachers, in order to carry out such a complex, emotionally and cognitively demanding job, must have continuous professional development of the highest possible quality and with the best possible working conditions, although this is not generally the case [6].

Due to this, and to the faster and unforeseen changes we have been subjected to in recent years, which have affected schools and education systems [7,8], including the evolution of science and technology, there is a need to develop and strengthen the links between training, teaching practice and educational research [9]. Several universities in different countries show a special interest in this issue, integrating it into institutional policy [10,11].

Until the end of the 20th century, researchers in the field of psychology and education believed that intellectual competencies were a crucial determinant of educational and occupational achievement [11] However, in the current century, soft skills, such as motiva-tion, teamwork, work ethic, planning, effective communication, and cultural awareness, are beginning to be considered to play an equally or more important role in school and work success. Currently, there is a greater awareness of the role of education in the promotion of soft skills and, therefore, new didactic methodologies for their instruction and new methods and instruments for their evaluation are being developed [12]

These issues also highlight the intellectual challenge required in the contemporary science classroom, the social nature of learning, the professional identity of teachers and the acquisition of a professional language related to learning and teaching. In addition, the personal resource input of trainee teachers is increasingly seen as a key factor for effectiveness, innovation and maintaining a healthier and more committed teaching staff [13].

Teacher competency frameworks and professional standards will define what is expected of teachers and what they are able to do, i.e., their pedagogical skills, which are considered key tools to encourage and support teacher quality [14]. These skills can serve as a basis for conceptualizing quality, assessing performance and developing teaching capacity [2,15]. Significant cognitive, social and affective outcomes have also been reported when learner-centered teaching strategies such as role-play, interactive learning, discovery learning, enquiry or differentiated learning and group work are applied, contributing effectively to the teaching and learning among children and young people [16,17].

In recent decades, so-called “soft skills” have started to be taken into account and they are being seen a necessary complement to traditional hard skills [18,19]. Hard skills are teachable and specific skills that can be defined and measured such as mathematics, writing, typing, etc.; while soft skills such as listening skills, personal habits, assertiveness, sensitivity, or getting along with others, are difficult to quantify [20]. Consequently, pure applied science is now less frequently considered in teacher education and instead, training programs seek to integrate and link this applied science with a reflective approach, which is seen as an increasingly critical component of education [21,22]. This type of experiential learning encourages students to make more stable connections between practical knowledge, theoretical knowledge and personal experience [22] and, in addition, it has been demonstrated to help self-efficacy promotion, self-esteem, responsible social awareness, beliefs, values, etc., which implies a holistic development of the learner and not just a mastery of purely didactic skills [23,24].

Globalisation and technological advances bring with them new ways of educating and, consequently, new approaches and methodologies that require the development of specific skills. This context is the reason to pay more attention to soft skills education.

As a novelty, this study tries to assess the personal skills of teachers (assertiveness, networking skills, teamwork, sensitivity, socialization, action orientation, ability to work under pressure and social desirability) and relate them to age, gender, nationality educational sector and digital skills. The sample is made up of Italian and Portuguese teachers, whose educational systems belong to the European tradition.

## 2. Materials and Methods

### 2.1. Study Design

An exploratory descriptive cross-sectional study was carried out in two groups of teachers, one in Italy and one in Portugal, in order to know comparatively their soft and digital skills profiles.

After agreeing to participate, each teacher answered the Business-focused Personality Inventory (BIP) questionnaire already adapted and used in the educational context [25] and received as feedback a personal profile indicating their interpersonal skills. The Italian sessions were conducted during the month of July 2021, the Portuguese sessions were conducted during the month of September 2021.

A focus group composed of experienced teachers and managers from both countries was interviewed within the research process to establish the premises that should be addressed in the discussion.

All participants accepted to participate and this research respected, at all times, the requirements established in the Declaration of Helsinki of 1975.

### 2.2. Study Participants

The sample is non-probabilistic and consists of 212 secondary school teachers, in particular, 106 Italian teachers (mainly from the area of Rome) and 106 Portuguese teachers (mainly from the area of Porto). The geographical distribution of the sample is due to the close collaboration between the Rome Business School and the Politechnic of Porto–Accounting and Business School (ISCAP). The Italian sample was composed of 28.3 % of women and 71.6 % of men, while the Portuguese group was composed of 64.1 % of women and 35.8 % of men. The age of the respondents in both groups was between 20 and 69 years (age M = 45.79). All participants indicated their agreement to participate in the Research. The university teachers from Rome Business School are working in a private organization; the Portuguese are from public and private polytechnic organizations.

### 2.3. Instruments

Each university teacher reported the Business-Focused Inventory of Personality [BIP] questionnaire and received a personal profile indicating his/her soft skills as feedback. This instrument aims to assess personality in the context of work, based on the theoretical bases of personalization and motivation and has been validated with a sample of 580 people so that the results pointed to a structure of three factors with alphas between 0.86 and 0.93 [26]. Questions were distributed over two sections: the first section corresponded to the socio demographic characteristics (gender, age and origin) and to the studies (University and type of degree). The second section corresponded to the self-evaluation of individual soft skills. The questionnaire comprises 14 scales, grouped in four domains plus the scale of impression management. In this case, six soft skills have been assessed: assertiveness, team orientation, sensitivity, action orientation, work under pressure, social desirability. The response is requested on a scale of six points that vary between ‘Completely true’ and ‘Completely false’. The variables studied are grouped into three areas: intra-personal, interpersonal and activity development. In addition, some questions focusing on impression management were presented. This scale indicates the tendency of the respondents to, consciously or unconsciously, give a positive and socially accepted image of oneself. Very extreme scores on this scale invalidate the complete questionnaire, since it is considered that the subject has not responded with sincerity. The questionnaires were distributed in two sections: the first one corresponds to socio-demographic characteristics (gender, age and origin) and to their studies (university and type of degree, highest degree they have, years of teaching experience, type of school they work in, years in the company, whether they have had an interview and prior relevant training or not). 

The second section corresponds to the self-assessment of soft skills. In total, the BIP questionnaire consists of 14 scales, grouped into four domains plus the impression man-agement scale. In this case, seven soft skills were self-assessed: assertiveness, networking skills, teamwork, sensitivity, action orientation, ability to work under pressure and social desirability [27], as is described in Table 1.

Responses were organized on an eight-point scale ranging from “Completely true” to “Completely false”. The variables were grouped into two areas: intrapersonal and inter-personal. Very extreme scores on this scale invalidate the entire questionnaire, as they indicate that the participant has not answered truthfully. 

The soft skills profile obtained in the two samples through the standardized BIP questionnaire is presented and compared with the normative score of the questionnaire. The focus group composed of experienced teachers and managers built the premises to be discussed in the conclusion of this research. The qualitative data collection aimed at proposing effective soft skills improvement strategies, methodology and research procedure that have high impact on values that become functional in organizational contexts where the ability to work in groups, the communication processes aimed at change management, and the culture of innovation are crucial. 

### 2.4. Statistical Analysis

The sample consisted of 212 teachers, 106 Italian and 106 Portuguese. The sample is balanced in both nationalities; however, the overall percentage is 71.6 % male in Italy and 64.1 % female in Portugal. Therefore, male teachers in Portugal and female teachers in Italy are slightly under-represented in the sample.

From the data, several types of comparisons and analyses were made with respect to skills. The soft skills levels of teachers were analyzed in relation to their age, gender, nationality and public/private sector. Soft skills covered assertiveness, networking, teamwork, sensitivity, socialization, action orientation, ability to work under pressure and so-cial desirability.

For the comparison between age and soft skills, the Kruskal Wallis test was used to measure the difference in the distributions of soft skills examined in relation to the age groups considered during the research. In addition, a comparison of the soft skills levels of each age group with all other age groups was carried out using Dunn’s test.

The soft skills that teachers possessed were then related to their digital skills. The level of digital skills of the group of teachers was measured using the Europass Online. 

Statistical analysis was performed with SPSS version 28 software (IBM-Inc, Chicago, IL, USA). A *p*-value < 0.05 was established for the analysis of statistical significance.

## 3. Results

As for the Italian teachers, there are no significant differences in the soft skills variable compared to the level of digital skills. Among Portuguese teachers, however, differences emerged, with older teachers being expert users (M: 79%) and basic-independent users being more able to network and more team-oriented than the Italian sample by age. 

Table 2 shows that the age profile of Italian teachers is somewhat higher than that of Portuguese teachers. In the 60–69 age classes the proportion of Italians is higher. However, this is also the case in the 50–59 and 30–39 age classes. In turn, Portuguese teachers dominate the age class 20 to 29.

Regarding the skills assessed by age groups between Italian and Portuguese teachers, most of them showed a higher score in the group of Italian teachers with statistically significant differences (*p* < 0.05).

In assertiveness and teamwork, Italian teachers scored higher in all age groups, not being significant between 20–29 years and 30–39 years, respectively. All age groups scored significantly higher on the ability to establish contact, sensitivity and ability to work under pressure. With regard to action orientation, Portuguese teachers scored significantly higher in the 40–49 and 60–69 age groups.

Socialization ability indicates a statistically significant difference with higher scores for Portuguese teachers in the 40–49 and 50–59 age groups, but it is in the 20–29 age group that higher scores are observed for Italians. Finally, social desirability shows significant differences with higher scores for Italians, but only in the 30–39 and 50–59 age groups (Table 3).

With regard to the assessment of skills according to the sector to which the teachers evaluated belonged, it could be observed that the Italians obtained statistically significant higher scores in most of them for both sectors (public and private); except for socialization skills where a significantly higher difference was found for the Portuguese (both in the public and private sectors) and in action-orientation, with a higher score in the private sector.

Social desirability showed no significant differences between Italians and Portuguese in either sector, and nor did action orientation in the public sector (Table 4).

Regarding sector (public/private) there are no significant differences except on Portuguese teachers on socialization skills, specifically on action-orientation skill, presenting a high score in the private sector.

Finally, Italian teachers also obtained statistically significant better overall scores in most skills taking into account gender. Only socialization stood out in favor of Portuguese teachers, with significantly higher scores for both genders, as well as in the action-orientation skill, where Portuguese men also stood out in terms of their scores. In the social desirability skill, men showed no statistically significant differences between the two nationalities, as applies also to women in the action-orientation skill (Table 5).

## 4. Discussion

This study indicates that teachers generally have high levels of interpersonal skills, despite the relatively small sample size. This may be due to or explained by the fact that teachers’ work is characterized by social interaction. The skills with the highest overall scores in this sample were assertiveness, networking, teamwork and sensitivity, with the lowest scores for both groups (Italian and Portuguese) being social desirability and action orientation. 

Perhaps teachers are increasingly in need of interpersonal skills due to the innovation and new types of creative networks emphasized in the EU modernization agenda; however, the most relevant skills mentioned in the study need to be examined closely to determine their importance in relation to the context. The comparison made between a sample of Italian and Portuguese teachers shows a fairly similar profile, although with some differences regarding certain skills: Portuguese teachers seem to be more sociable and action-oriented, while Italians show higher levels of assertiveness, networking skills, teamwork, sensitivity and ability to work under pressure.

The categories of intrapersonal skills and interpersonal skills, according to Boyatzis et al. [34], align with the main elements of emotional intelligence. This gives a reason to consider an approach that covers a broader consistency between inter/intrapersonal skills and nations/cultures. 

The WEF study (2018) entitled “Eight Futures of Work: Scenarios and their Implica-tions”, presents several possible visions of what the future of work could be like until 2030, stating that “(…) the evolution of learning between the current and future labour force; and the magnitude of talent mobility between geographies, are likely to influence the nature of work in the future (…)” [35].

In addition to cultural factors, the reported differences in skill levels can be traced to the different educational systems and cultures prevailing in the two countries. 

Even if both countries encourage higher education for teachers, the Italian system is based on a general consistency of approach and obligatory requirements in the teacher education system, while the Portuguese system traditionally relies more on the independence of particular educational institutions, especially in higher education. It is most likely that different types of skills have gained special attention in the two systems. These factors may be visible in the official curricula, or they may be more invisible in the hidden curricula, although they are strongly connected to learning methods and processes.

This study suggests that Portuguese teachers have reasons to improve their soft skills in the intrapersonal area, meaning assertiveness and aspects of teamwork; and in the interpersonal area, sensitivity and networking. Italian teachers should work on socialization and action orientation.

However, it should be mentioned that the sample size does not allow for very strict conclusions, so the study must be considered within its limitations and results must be taken into account cautiously. However, several of the conclusions and suggestions presented motivate to deepen the study with larger samples and with a more homogeneous sample with respect to gender; this could generate data on soft skills that are fundamental when it comes to renewing teacher education and in-service training.

It is worth highlighting the difference between skills, given the different challenges of including soft skills in teacher training and curricula in addition to the already present hard skills, which are of a totally different character. Soft skills, being related to the person and the context, can be changeable and adaptable to different situations, which gives them a subjective character and makes them difficult to measure and assess. Therefore, the scores obtained in the study regarding the rating of the level of skills used are based on a self-assessment, which does not provide as in-depth results as in the 360-degree type of assessment, but is easier to organize as a research constellation; it is necessary to create appropriate assessment frameworks for the measurement of soft skills.

It is important that qualitative results integrate data derived from quantitative results and consider the complexity of the teacher’s role in 2020.

Attempts are currently being made to discover the essential qualities of a good teacher around the world, although trying to put them into words is a difficult task; researchers have produced lists of competences, strongly supported by policy makers [36]. Some researchers point out the impossibility of defining “good teaching” with normative frameworks, noting that these definitions are variable depending on the context and individual teacher characteristics [37]. Others deny the definition of teaching standards, stressing that this ignores the complexity of the profession, while most agree that they should be based on effective teaching practice, based on values and beliefs, respecting the aims of teaching, and respecting the objectives of the profession [38,39].

Stakeholder involvement can help ensure that standards are owned by the profession rather than imposed as a top-down approach to teacher accountability [40]; at the same time, broad participation in the design of competency frameworks and these professional standards can encourage the acceptance and assurance of those with different views and experiences [37].

Competency frameworks and professional standards define what teachers are able to do and what they should know how to do, avoiding the methodological challenges of measuring teacher quality with “proxy measures” such as years of professional experience or student performance. These measures do not report on support for student development or the ability to create learning opportunities. [41] Competency frameworks and professional standards are meant to be pragmatic tools, based on functional analyses of teachers’ tasks, and supporting the expected adherence to the country’s codes of ethics and cultural values [42]. At the same time they are aligned with National Qualification Frameworks that reflect the formal learning and certification required in various sectors and specific jobs, including education [16,18].

The educational contexts are changing accordingly. They require skills in multi-channel communication, facilitating interaction, flexibility and use of different tools (Horizon, 2014 and subsequent publications). There are calls for the adoption of open innovation mindset, framework 4.0, social technologies, digital strategies, and other enabling technologies [18].

Therefore, the teacher education curriculum process obviously needs to be able to provide relevant knowledge and skills, as well as conceptual and soft skills in addition to hard skills. This inclusion of soft skills within the curriculum can ensure the success of the profession in the 2020s, but in order for teachers to be able to adapt to innovations, changes and be informed about developments in education, they should be prepared for professional development as educators throughout their careers. To this end, in-service training courses (INSET) have an essential role to play in teaching teachers about innovations and changes with the aim of facilitating change [43]. 

These online programs are being organized as an empowering solution to ensure teachers’ professional development in this era of digitalization [44]; although some research indicates that teaching in the way they were taught, using purposeful prior practice, may also be an option, and may be positive [45].

Reviews of the development of instruction in higher education [46,47] have argued for greater variability in methodology and approaches in researching the impact of development initiatives by measuring actual behavioral outcomes. In this way, at least the common pitfalls of traditional self-report questionnaires, such as the difficulty in detecting unconscious processes [48] and the risk of socially desirable responses, can be avoided [49]. 

As a limitation, it is worth mentioning that the changes have been measured by interpretations of the various questions, and may not reflect the performance of the teachers who have been surveyed. However, previous research mentions the fact that conceptions can be related to approaches [50], so it may be likely that interpretations are also connected to teachers’ approaches.

## 5. Conclusions

Teaching is a complex act, requiring a wide range of knowledge and skills to success-fully manage the demands of the classroom [42,51]. Therefore, the importance of related training and strengthening of these skills in university teachers should not be underestimated and will make a difference to the quality of effective teaching and learning [52]. While there is evidence supporting the importance of soft skills such as communication, creative thinking, problem solving and teamwork in university teaching practice [53], it is considered essential to raise awareness in society and among university teachers themselves about the needs and expectations of these requirements and encourage demand for action in the context of higher education.

The teaching curriculum that has been proposed in each new reform has been introducing novel topics that increasingly open the way for school practice and explicitly for educational research; but the division of time, knowledge and space still remains unexplored. 

In order to achieve these fundamental changes, a curriculum-centered teaching and learning of soft skills, using them as concrete methodological approaches and not as abstract pedagogical guidelines, has been promised as a vehicle. This approach therefore highlights the importance of pedagogy as a strategic point in the education system.

As a final conclusion, it is important to consider the incorporation of hard and soft skills courses in the teaching curriculum in order to motivate teachers and improve the values of humanistic pedagogy, which would promote the pedagogical culture and generate forms of continuous learning.

## Figures and Tables

**Table 1:** Definitions of the skills used the study.

Skill	Definition
Assertiveness	Ability to stand up for one’s own or others’ rights in a calm and positive manner, without being aggressive or passively accepting “the bad” [28].
Capacity for networking	It consists of a variety of skills related to communication, openness and the ability to create a mutual experience of trust in other people
Teamwork	Qualities and skills that enable one to work well with others during conversations, projects, meetings, etc.; it depends on the ability to communicate well, listen actively, and be responsible and honest [29].
Sensitivity	Ability to notice things and care about people’s feelings; taking time to assimilate and process, rather than rushing to a decision [30].
Action oriented	A bipolar dimension: action orientation favors the transformation of intention into action. In contrast, state orientation is characterized by having thoughts in the mind related to the achievement of a goal [31].
Ability to work under pressure	Self-image in the ability to perform their duties despite the circumstances, maintaining a constant level of efficiency. [32].
Social desirability	Used as a control factor in the study: type of response bias which is the tendency of respondents to answer questions in a way that will be viewed positively by others [33].

**Table 2:** Age range by nationality.

			Age Range				
			20–29	30–39	40–49	50–59	60–69
Nationality	Ita	% within the age range	3.88%	25.2%	33.0%	34.9%	6.79%
	Pt	% within the age range	11.6%	17.4%	33.9%	33.0%	3.88%

Ita: Italian; Pt: Portuguese.

**Table 3:** Self-assessment of age-related competences.

				PT			ITA		
		N	Mean	St. Dev	N	Mean	St. Dev	*t*-Test	*p*-Value
AS	20–29	12	5.5	0.57	4	6	1.15	−0.42	0.34
	30–39	18	5	0.2	26	6.38	0.28	−3.52	<0.001
	40–49	35	4.54	0.16	34	5.65	0.26	−3.67	<0.001
	50–59	34	4.41	0.25	36	5.78	0.29	−3.57	<0.001
	60–69	4	5.5	0.29	4	7.5	0.29	−4.9	0.001
CN	20–29	12	3.67	0.28	4	7.5	0.87	−5.6	<0.001
	30–39	18	3.44	0.12	26	5.62	0.36	−4.93	<0.001
	40–49	35	3	0.26	34	5.47	0.34	−5.79	<0.001
	50–59	34	4.65	0.34	36	6.39	0.29	−3.92	<0.001
	60–69	4	3	0.58	4	8.5	0.29	−8.52	<0.001
TW	20–29	12	5	0.43	4	7.5	0.29	−3.24	0.003
	30–39	18	4.61	0.23	26	5.38	0.38	−1.53	0.067
	40–49	35	3.85	0.23	34	5.94	0.34	−5.08	<0.001
	50–59	34	4.21	0.17	36	6.67	0.27	−7.51	<0.001
	60–69	4	4.5	0.29	4	8.5	0.29	−9.8	<0.001
SN	20–29	12	3.17	0.56	4	8	0	−4.82	<0.001
	30–39	18	2.61	0.35	26	5.31	0.49	−4.07	<0.001
	40–49	35	2.29	0.17	34	5.71	0.31	−9.75	<0.001
	50–59	34	2.32	0.24	36	6.17	0.27	−10.73	<0.001
	60–69	4	2	0.58	4	8.5	0.29	−10.07	<0.001
SO	20–29	12	3.83	0.11	4	4.85	0.29	−2.65	0.01
	30–39	18	3.78	0.33	26	3.31	0.24	1.18	0.12
	40–49	35	4.57	0.24	34	3.17	0.14	4.96	<0.001
	50–59	34	5.26	0.16	36	3.72	0.15	7.12	<0.001
	60–69	4	4	0.58	4	4	0	0	0.5
AO	20–29	12	3.17	0.41	4	3	0	0.23	0.41
	30–39	18	2.56	0.31	26	2.15	0.13	1.31	0.09
	40–49	35	2.94	0.16	34	2.29	0.11	3.29	<0.001
	50–59	34	2.29	0.17	36	2.39	0.1	−0.49	0.31
	60–69	4	2.5	0.29	4	1.5	0.29	2.45	0.025
AUP	20–29	12	3.33	0.41	4	7.5	0.29	−5.55	<0.001
	30–39	18	3.61	0.16	26	5.61	0.42	−3.25	0.001
	40–49	35	2.97	0.15	34	6.12	0.33	−8.84	<0.001
	50–59	34	3.09	0.19	36	5.95	0.21	−10.12	<0.001
	60–69	4	2.5	0.87	4	7	1.15	−3.12	0.01
SD	20–29	12	3.5	0.38	4	2.5	0.87	1.22	0.12
	30–39	18	2.33	0.28	26	2.92	0.2	−1.76	0.042
	40–49	35	2.49	0.26	34	2.94	0.21	−1.36	0.09
	50–59	34	2.29	0.2	36	2.78	0.18	−1.78	0.039
	60–69	4	2.5	0.87	4	3.5	0.29	−1.09	0.16

PT: Portuguese; ITA: Italian; AS: assertiveness; CN: capacity for networking; TW: teamwork; SN: sensitivity; SO: socialization; AO: action oriented; AUP: ability to work under pressure; SD: social desirability.

**Table 4:** Self-assessment of competences according to the sector.

				PT			ITA		
		N	Mean	St. Dev	N	Mean	St. Dev	*t*-Test	*p*-Value
AS	Public	37	4.81	0.13	16	5.37	0.26	−2.14	0.018
	Private	69	4.65	0.18	90	6.04	0.17	−5.52	<0.001
CN	Public	37	3.68	0.24	16	6	0.39	−5.24	<0.001
	Private	69	3.68	0.21	90	6.04	0.21	−7.88	<0.001
TW	Public	37	4.19	0.16	16	6.62	0.36	−7.08	<0.001
	Private	69	4.2	0.17	90	6.15	0.21	−6.98	<0.001
SN	Public	37	2.27	0.2	16	5.25	0.59	−6.05	<0.001
	Private	69	2.52	0.17	90	6.13	0.2	−13.16	<0.001
SO	Public	37	4.59	0.21	16	3.37	0.22	3.46	<0.001
	Private	69	4.51	0.16	90	3.51	0.1	5.49	<0.001
AO	Public	37	2.24	0.15	16	2.5	0.13	−1.07	0.145
	Private	69	2.87	0.14	90	2.22	0.07	4.42	<0.001
AUP	Public	37	3.08	0.17	16	6	0.46	−7.31	<0.001
	Private	69	3.22	0.13	90	5.87	0.19	−10.75	<0.001
SD	Public	37	2.24	0.23	16	2.75	0.33	−1.21	0.11
	Private	69	2.64	0.16	90	2.91	0.11	−1.45	0.074

PT: Portuguese; ITA: Italians; AS: assertiveness; CN: capacity for networking; TW: teamwork; SN: sensitivity; SO: socialization; AO: action oriented; AUP: ability to work under pressure; SD: social desiderability.

**Table 5:** Self-assessment of competences according to gender.

				PT			ITA		
		N	Mean	St. Dev	N	Mean	St. Dev	*t*-Test	*p*-Value
AS	Male	38	4.32	0.21	76	4.72	0.2	−4.78	<0.001
	Female	68	4.93	0.15	30	6.27	0.22	−5.02	<0.001
CN	Male	38	3.74	0.27	76	6.03	0.23	−6.18	<0.001
	Female	68	3.65	0.2	30	6.07	0.32	−6.54	<0.001
TW	Male	38	4.32	0.25	76	6.34	0.24	−5.36	<0.001
	Female	68	4.13	0.13	30	5.93	0.26	−6.81	<0.001
SN	Male	38	2.58	0.28	76	5.87	0.25	−8.13	<0.001
	Female	68	2.35	0.13	30	6.33	0.26	−14.92	<0.001
SO	Male	38	4.92	0.22	76	3.58	0.11	6.01	<0.001
	Female	68	4.32	0.15	30	3.27	0.16	4.3	<0.001
AO	Male	38	2.29	0.21	76	2.21	0.08	3.83	<0.001
	Female	68	2.5	0.12	30	2.4	0.11	0.52	0.3
AUP	Male	38	3.37	0.17	76	5.74	0.21	−7.22	<0.001
	Female	68	3.06	0.13	30	6.27	0.29	−11.72	<0.001
SD	Male	38	2.92	0.2	76	2.89	0.13	0.11	0.455
	Female	68	2.26	0.17	30	2.87	0.19	−2.15	0.02

PT; Portuguese; ITA: Italian; AS: assertiveness; CN: capacity for networking; TW: teamwork; SN: sensitivity; SO: socialization; AO: action oriented; AUP: ability to work under pressure; SD: social desirability.
